# Dataset of functional connectivity during cognitive control for an adult lifespan sample

**DOI:** 10.1016/j.dib.2021.107573

**Published:** 2021-11-15

**Authors:** Jenny R. Rieck, Giulia Baracchini, Daniel Nichol, Hervé Abdi, Cheryl L. Grady

**Affiliations:** a Rotman Research Institute at Baycrest, 3560 Bathurst Street, Toronto, ON M6A2E1, Canada; b Montreal Neurological Institute, McGill University, Montreal, QC, Canada; c The University of Texas at Dallas, Richardson, TX, USA; d Departments of Psychiatry and Psychology, University of Toronto, Toronto, ON, Canada

**Keywords:** *f*MRI, Normal aging, Functional connectivity, Cognitive control, Multivariate methods

## Abstract

We provide functional connectivity matrices generated during functional magnetic resonance imaging (*f*MRI) during different tasks of cognitive control in healthy aging adults. These data can be used to replicate the primary results from the related manuscript: *Reconfiguration and dedifferentiation of functional networks during cognitive control across the adult lifespan* (Rieck et al., 2021). One-hundred-forty-four participants (ages 20–86) were scanned on a Siemens 3T MRI scanner while they were completing tasks to measure functional activity during inhibition, initiation, shifting, and working memory. Estimates of functional connectivity (quantified with timeseries correlations) between different brain regions were computed using three different brain atlases: Schaefer 100 parcel 17 network atlas (Schaefer et al., 2018; Yeo et al., 2011), Power 229 node 10 network atlas (Power et al., 2011), and Schaefer 200 parcel 17 network atlas (Schaefer et al., 2018; Yeo et al., 2011). The resulting functional connectivity correlation matrices are provided as text files with this article. Cov-STATIS (Abdi et al., 2012; a multi-table multivariate statistical technique; https://github.com/HerveAbdi/DistatisR) was used to examine similarity between functional connectivity during the different domains of cognitive control. The effect of aging on these functional connectivity patterns was also examined by computing measures of “task differentiation” and “network segregation.” This dataset also provides supplemental analyses from the related manuscript (Rieck et al., 2021) to replicate the primary age findings with additional brain atlases. Cognitive neuroscience researchers can benefit from these data by further investigating the age effects on functional connectivity during tasks of cognitive control, in addition to examining the impact of different brain atlases on functional connectivity estimates. These data can also be used for the development of other multi-table and network-based statistical methods in functional neuroimaging.

## Specifications Table

**Table:** 

Subject	Neuroscience: Cognitive
Specific subject area	Functional neuroimaging, task-based functional connectivity, aging, cognitive control
Type of data	Tables Figures Functional connectivity correlation matrices Code
How data were acquired	Task-based functional magnetic resonance imaging (3T Siemens scanner)
Data format	Analyzed
Parameters for data collection	Blood-oxygen-level-dependent (BOLD) *f*MRI data were collected using an echo-planar imaging sequence with 40 axial slices acquired parallel to the anterior-posterior commissure with the following parameters: TR = 2000 ms, TE = 27 ms, Flip Angle = 70°; FOV = 192 mm, 64 × 64 × 40 acquisition matrix; 3 mm^3^ isotropic voxels (with .5 mm gap). Timeseries correlations were used to quantify functional connectivity for three different brain atlases.
Description of data collection	144 healthy adults (ages 20–86) underwent *f*MRI while completing three tasks to measure different domains of cognitive control. A go/no-go paradigm was used to examine inhibition and initiation. A local task switching paradigm was used to examine shifting. An n-back paradigm with three different loads (0-back, 1-back, and 2-back) was used to examine working memory.
Data source location	Institution: Rotman Research Institute, Baycrest Center City/Town/Region: Toronto, Ontario Country: Canada
Data accessibility	Repository name: Dataset of functional connectivity during cognitive control for an adult lifespan sample Data identification number: 10.17605/OSF.IO/M5CRS Direct URL to data: https://doi.org/10.17605/OSF.IO/M5CRS
Related research article	J.R. Rieck, G. Baracchini, D. Nichol. H. Abdi, C.L. Grady, Reconfiguration and dedifferentiation of functional networks during cognitive control across the adult lifespan. *Neurobiology of Aging*. 2021. https://doi.org/10.1016/j.neurobiolaging.2021.03.019

## Value of the Data

• These data can be used to replicate the primary analyses of Rieck and colleagues [1] across three different functional connectivity brain atlases [2], [3], [4].
• Researchers can benefit from these data by investigating age effects on functional connectivity during tasks of cognitive control.
• These data can be analyzed to further understand the impact of different brain atlases on functional connectivity estimates.
• These data can also be used for the development of multi-table and network-based statistical methods in functional neuroimaging.

## 1 Data Description

The data provided with this manuscript are text files of functional connectivity correlation matrices generated from functional magnetic resonance imaging (*f*MRI) during tasks of cognitive control (https://doi.org/10.17605/OSF.IO/M5CRS). There are six correlation matrices per participant, a design corresponding to six different task conditions: inhibition (*gng_inhibition.txt), initiation (*gng_initiation.txt), shifting (*tsw_shifting.txt), and working memory at three loads, 0-back (*nbk_0back.txt), 1-back (*nbk_1back.txt), and 2-back (*nbk_2back.txt).

Functional connectivity correlation matrices are provided for three different parcellation atlases: (1) Schaefer 100 parcel, 17 network atlas [2,3] (Schaefer100_17*) used for the primary analyses in the related research article [1]; (2) Schaefer 200 parcel, 17 network atlas [2,3] (Schaefer200_17*); and (3) an iteration of 229 nodes provided by Power and colleagues for 10 networks [4] (Power229_10*). The Schaefer200_17 and Power229_10 atlases were used to repeat the primary analyses in the related research article [1] in order to see if the effects replicated (results reported here).

In addition to functional connectivity matrices, we provide a .csv file of participant ids and corresponding ages (*participant_ages.csv*) and .csv files that include centroid coordinates (*Power_229node_10network_coordinates.csv*) and anatomical labels for the parcellation atlases (*Schaefer_100parcel_17network_labels.csv, Schaefer_200parcel_17network_labels.csv*). We also include e-prime scripts to replicate the fMRI experiments and an R script (*run_covstatis.R*) and functions (*fc_data_in.R, compute_task_diff.R, compute_segregation.R*) to reproduce the primary statistical analyses, compute task differentiation and segregation composite measures, and recreate plots presented in the related research article [1] and current manuscript.

Finally, provided in this manuscript are several tables and figures of the secondary analyses of this dataset, including:

• Table 1. *Summary of original findings and additional atlas* analyses*.*

**Table 1:** Summary of original findings and additional atlas analyses.

Schaefer 100	Power 229	Schaefer 200
Original Finding	Finding Replicated?	Finding Replicated?
*A. Age-related decreases in task differentiation*		
Control	Yes	Yes
Default Mode	No	Yes
Salience/Ventral Attention A	Yes (for CingOp)	Yes
Temporo-Parietal	Yes (for VAN)	No
Somato-Motor	No	Yes
*B. Age-related decreases in network segregation*		
Control	Yes	Yes
Default Mode	Yes	Yes
Somato-Motor B	Trend (for AUD)	Yes
Salience/Ventral Attention A	Yes	No
Dorsal Attention	Yes	Yes
*C. Age-related increases in network segregation*		
Somato-Motor A	Yes	Yes
Salience/Ventral Attention B	No (for SAL)	No

The first column outlines the significant age findings by network using the Schaefer100_17 atlas. Columns two and three indicate which effects were replicated (*p* < .05) or reduced to trend effects (*p* < .1). For the Power229_10 atlas, the closest equivalent network is indicated in parentheses. *Note*. CingOp = Cingulo-opercular; VAN = Ventral Attention; AUD = Auditory; SAL = Salience.
• Table 2. *Overlap between Schaefer 100 and Power 229 atlases.*

**Table 2:** Overlap between Schaefer 100 (rows) and Power 229 atlases (columns).

		Power Atlas (229 5 mm spherical nodes, 10 networks)									
		VIS	SomM	AUD	DAN	CingOp	SAL	VAN	FPC	DMN	SUB
**Schaefer Atlas 100 parcel, 17 network**	Visual A	27.7								<5	
	Visual B	**28.4**								<5	
	SomMot A		**47.0**			<5				<5	
	SomMot B		10.5	**57.7**		<5		12.5		<5	
	TempPar			<5				**40.3**		<5	
	DAN A	15.3			**51.1**				9.5	<5	
	DAN B		13.9	8.2	39.2	11.2	<5	<5	<5		
	Sal/VAN A		<5	15.9		**48.2**	14.9	6.9	<5	<5	<5
	Sal/VAN B					7.6	**38.2**		8.0	<5	
	Control A		<5		<5	<5	<5	<5	**39.5**		
	Control B						<5		21.5	5.9	
	Control C	<5	<5							<5	
	Default A	<5					5.6	<5	<5	21.0	
	Default B			<5			10.4	21.5	9.3	**32.1**	
	Default C	<5								<5	
	Limbic A								<5	<5	
	Limbic B										

**Fig. 1:**
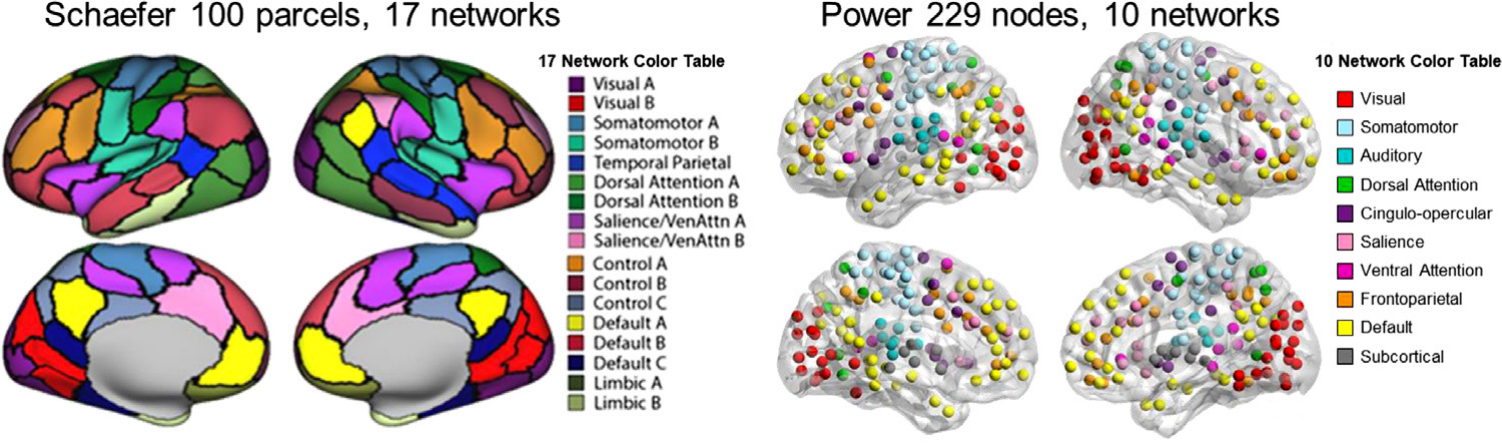
*Schaefer and Power Atlas Comparison.* Comparison of Schaefer 100 parcel, 17 network (left) and Power 229 node, 10 network (right) atlases. Modified from [1] (Fig. S2).
• Fig. 2*. Power Atlas Multivariate Connectivity Space.*

Values represent the percentage of overlap of voxels from each of the ten Power atlas networks and the corresponding Schaefer networks. For example, 27.7% of voxels in the Power Visual network overlap with Schaefer Visual A and 28.4% of voxels in the Power Visual network overlap with Schaefer Visual B. *Note*: VIS = Visual; SomM = Somato-Motor; AUD = Auditiory; DAN = Dorsal Attention; CingOp = Cingulo-opercular; SAL = Salience; VAN = Ventral Attention; FPC = Fronto-parietal Control; DMN = Default Mode; SUB = Subcortical.
• Fig. 1. *Schaefer and Power Atlas Comparison.*

**Fig. 2:**
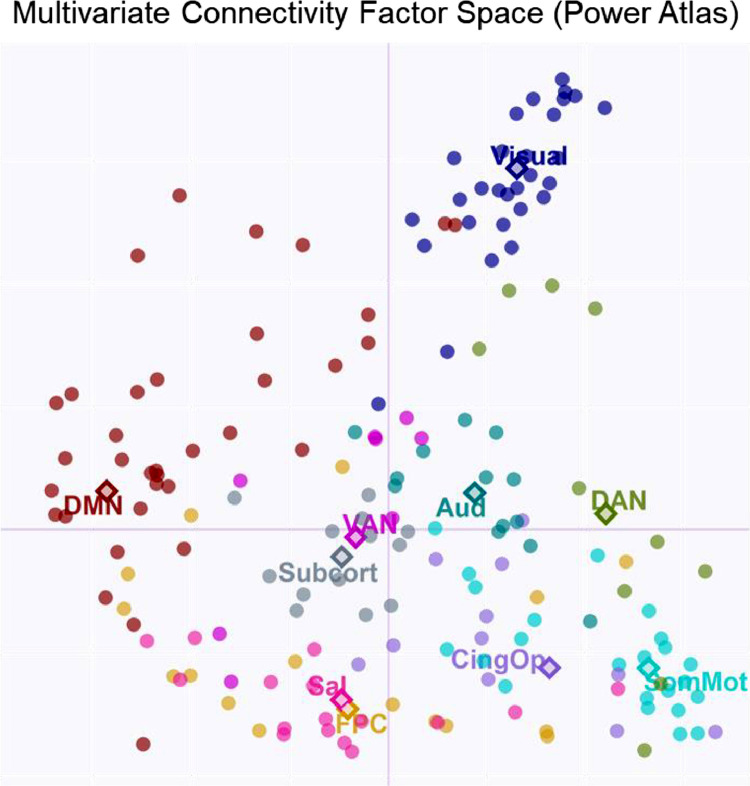
*Power Atlas Multivariate Connectivity Space.* Individual nodes (dots) from the Power229_10 atlas are projected in the compromise space (and colored by network).
• Fig. 3. *Power Atlas Task Differentiation and Age.*

**Fig. 3:**
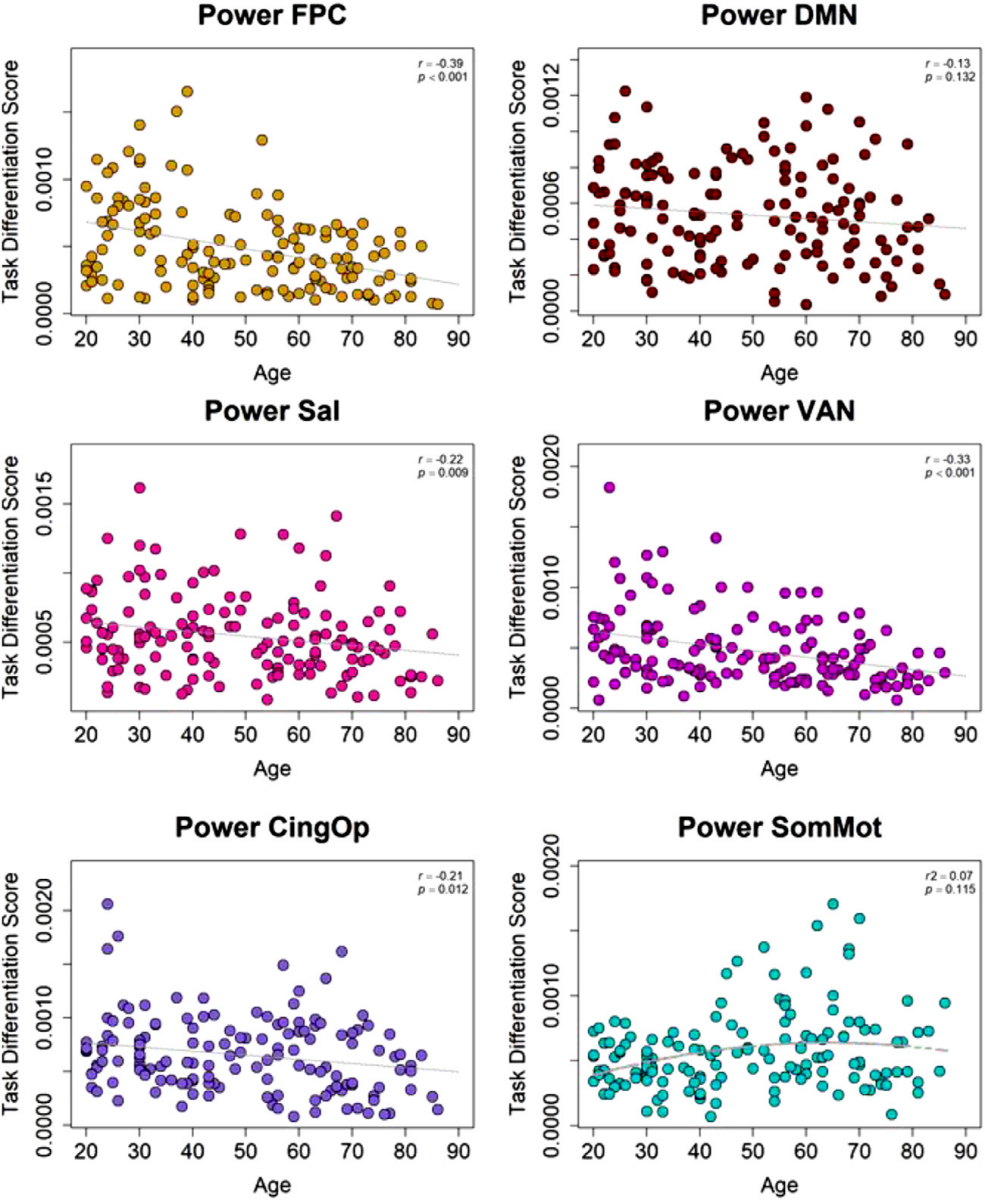
*Power Atlas Task Differentiation and Age.* Task differentiation scores for the Power fronto-parietal control, salience, ventral attention, and cingulo-opercular networks decreased in older ages. No age effect was replicated for the default or somato-motor network.
• Fig. 4. *Power Atlas Network Segregation with Age.*

**Fig. 4:**
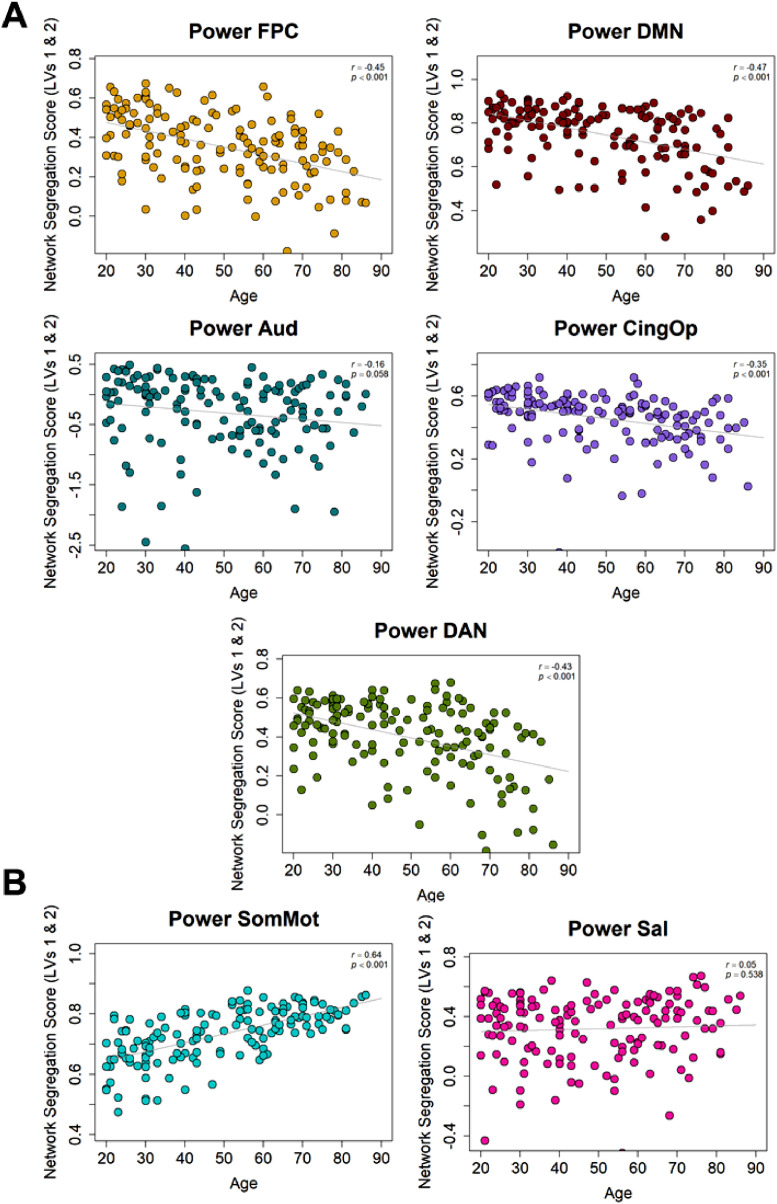
*Power Atlas Network Segregation with Age*. (A) Age-related decreases in network segregation were replicated for Power fronto-parietal control, default mode, auditory, cingulo-opercular, and dorsal attention networks. (B) Age-related increases in network segregation were replicated for the Power somato-motor network, but not the Power salience network.
• Fig. 5. *Schaefer 100 and 200 Parcel Atlas Comparison.*

**Fig. 5:**
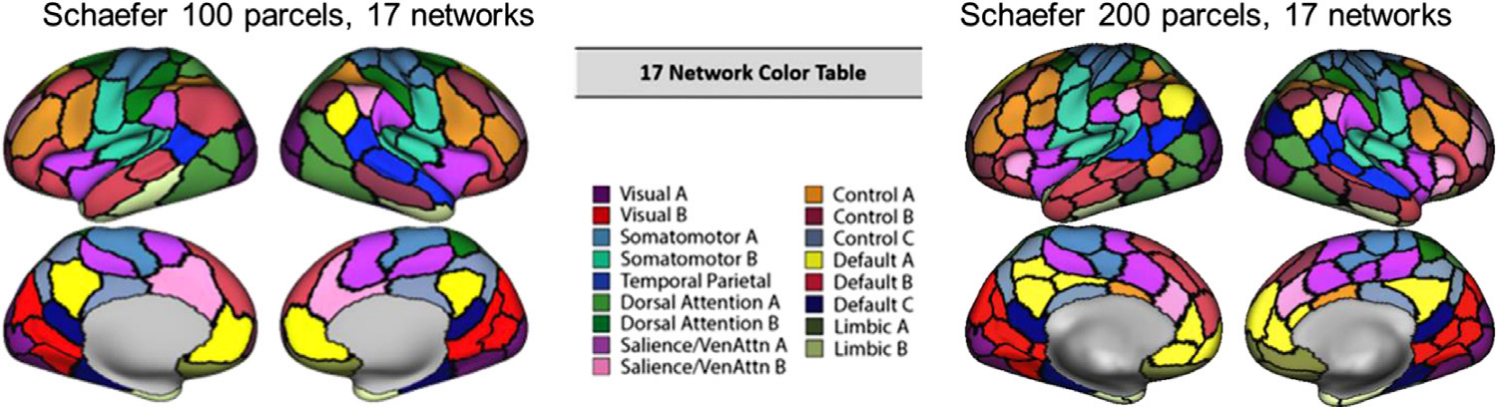
*Schaefer 100 and 200 Parcel Atlas Comparison.* Comparison of Schaefer 100 parcel (left) and 200 parcel (right) atlases. Modified from [1] (Fig. S2).
• Fig. 6*. Schaefer 200 Atlas Multivariate Connectivity Space.*

**Fig. 6:**
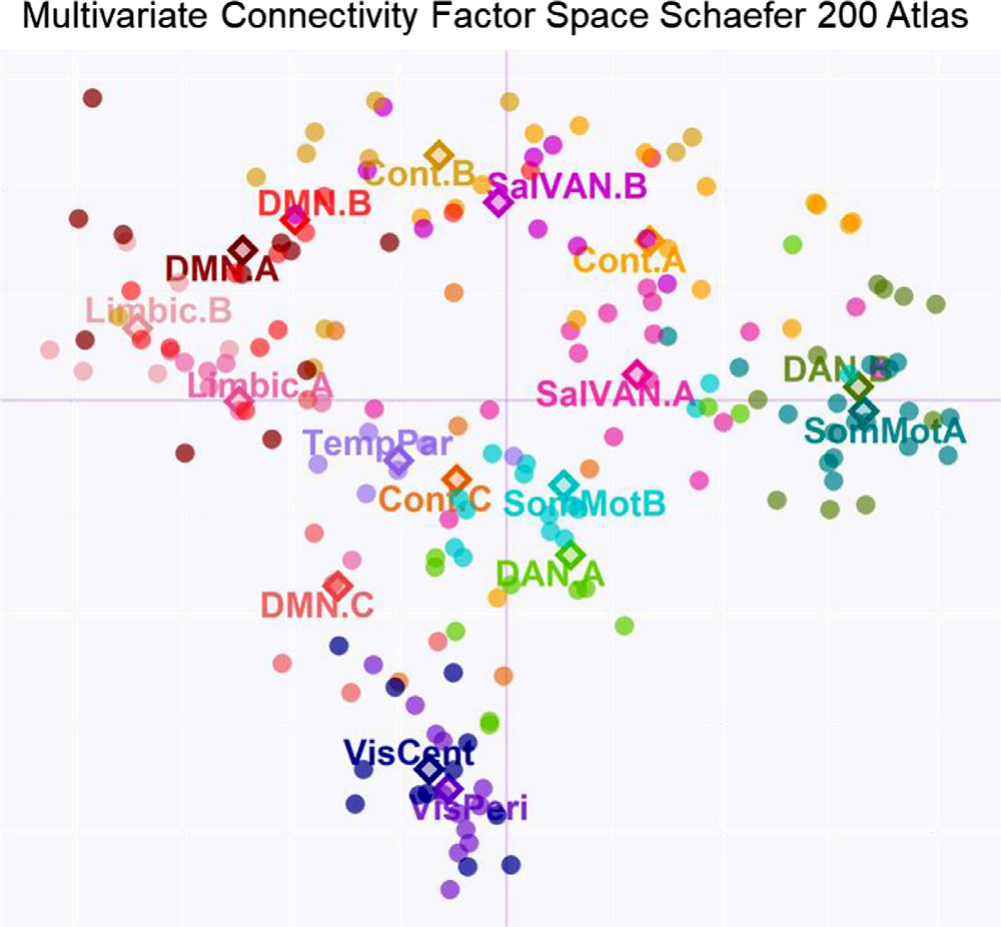
*Schaefer 200 Atlas Multivariate Connectivity Space.* Individual nodes (dots) from the 200 parcel atlas are projected in the compromise space (and colored by network).
• Fig. 7. *Schaefer 200 Atlas Task Differentiation and Age.*

**Fig. 7:**
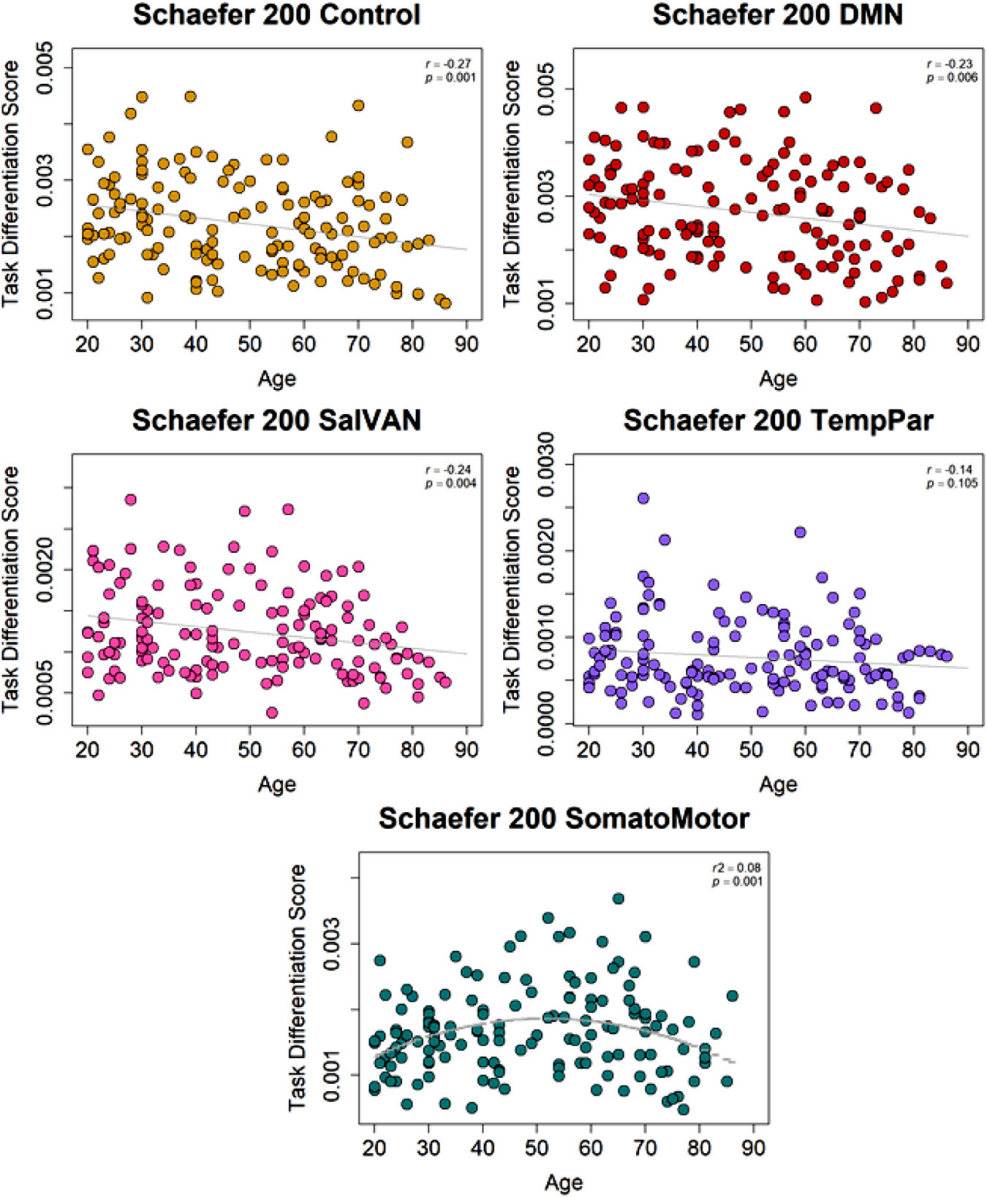
*Schaefer 200 Atlas Task Differentiation and Age.* Task differentiation scores for the Power fronto-parietal control, default, salience/ventral attention networks decreased in older ages, but no age effect on temporo-parietal was replicated. Age was also associated with non-linear effects on differentiation of the somato-motor network.
• Fig. 8. *Schaefer 200 Atlas Network Segregation with Age.*

**Fig. 8:**
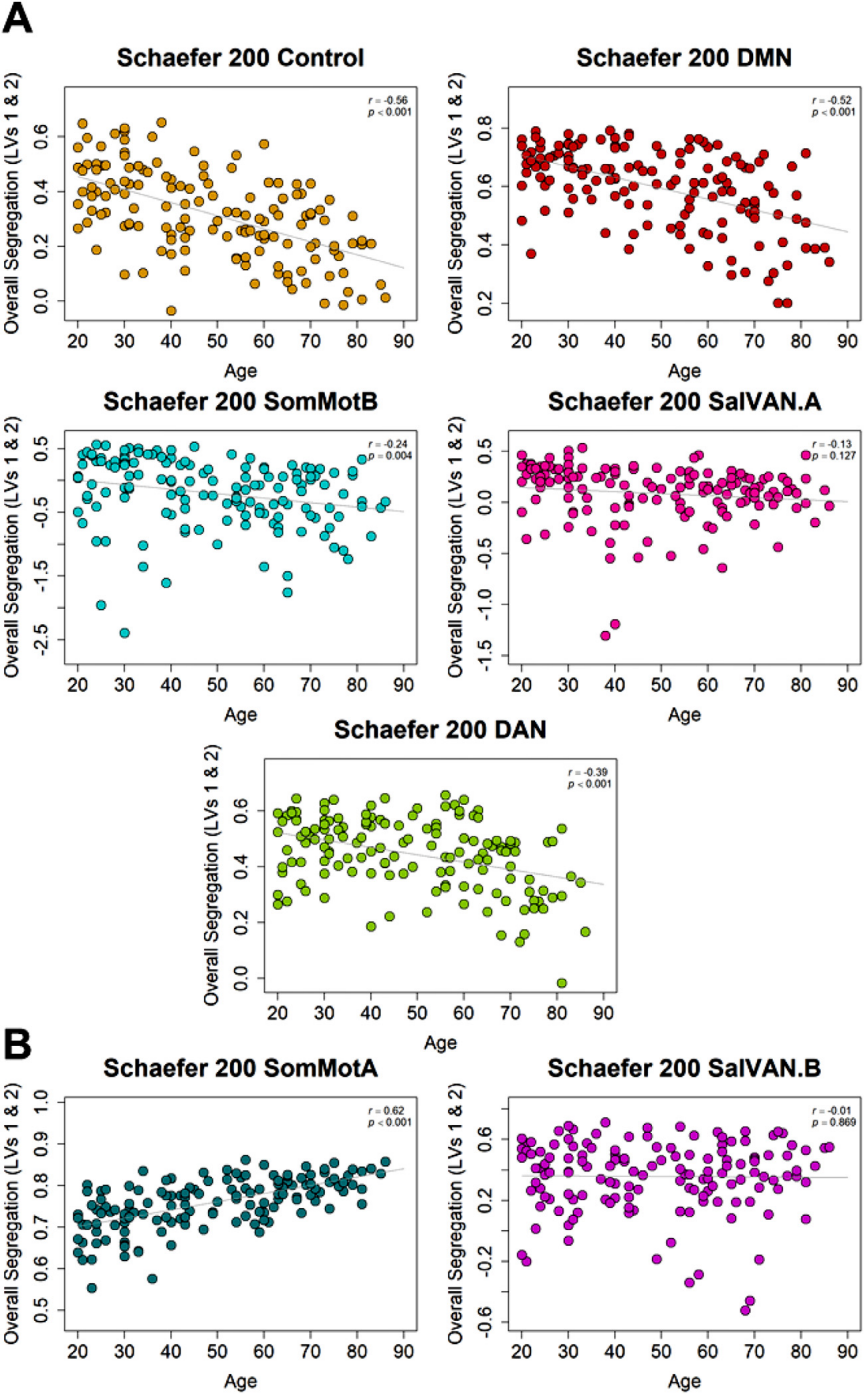
*Schaefer 200 Atlas Network Segregation with Age*. (A) Age-related decreases in network segregation were replicated for control, default mode, somato-motor B, and dorsal attention networks, but not salience/ventral attention A network. (B) Age-related increases in network segregation were replicated for the somato-motor A, but not the salience/ventral attention B network.

### 1.1 Supplemental analyses with alternate atlases

In order to investigate whether the primary findings [1] were driven by the original atlas choice (Schaefer atlas with 100 parcels in 17 networks), we re-ran the primary analyses using two additional atlases: Power atlas (229 nodes in 10 networks) and Schaefer (200 parcels in 17 networks). The Power atlas allowed us to examine broad networks that were defined differently from the original analyses. The Schaefer 200 parcel atlas allowed us to examine the same original 17 networks, but using a finer-grained approach with smaller parcels. In general, our original findings of age effects on task differentiation and network segregation were replicated across both atlases, with the exception of some findings associated with the salience/ventral attention network. Table 1 summarizes the original findings [1] and if they were replicated in the current work using different atlases.

As in the primary manuscript [1], we utilized a multivariate multi-table method of Procrustean analysis [5] called cov-STATIS, a (French) acronym which loosely translates to “structuring three-way statistical (in our case, correlation) tables” [6,[7], [8], [9] to examine the similarity of the functional network connectivity across task conditions. The *DistatisR* R package [10] was used to run this analysis separately for the three different atlas parcellations. The related research article [1] includes a detailed description on how this method was applied to functional connectivity data, and code to replicate the analyses is available (https://osf.io/atkb4/). The results from cov-STATIS were used to compute “task differentiation” scores which quantified how similar a participant's connectivity for a particular network was across all the six task-conditions and “segregation scores” which quantified relative strength of connectivity within a network versus between networks [11]. Both task differentiation and segregation scores were correlated with age to see if the primary findings from the related article [1] replicated when using different atlas parcellations.

#### 1.1.1 Power 229 atlas analyses and results

Using CONN, functional time courses for each task condition were extracted from 229 nodes that comprised 10 networks: somato-motor (SomMot), auditory (Aud), cingulo-opercular (CingOp), default mode (DMN), visual, frontoparietal control (FPC), salience (Sal), subcortical, ventral attention (VAN) and dorsal attention (DAN). Unlike the Schaefer atlas, which defines regions of interest/parcels that cover larger regions of cortex, for the Power atlas nodes were defined by creating 5mm spheres around the coordinates provided by Power et al. [4]. The coordinates for the nodes used for the current dataset are provided in *Power_229node_10network_coordinates.csv.*

The Power atlas also differs from the Schaefer 17 network atlas in how networks are defined (Fig. 1)—most notably Power provides broad network definitions, whereas the Schaefer 17 network atlas divides larger networks (e.g., default) to multiple subnetworks (e.g., default A, B, and C). The Power atlas also includes the salience and cingulo-opercular networks as two separate networks, whereas the Schaefer atlas combines the salience and ventral attention networks. The Schaefer salience/ventral attention A subnetwork corresponds to the Power cingulo-opercular network. Finally, the Power atlas defines a subcortical network, whereas no similar network exists within the Schaefer atlas. Table 2 shows the proportion of voxels from each Power network that overlap with the Schaefer100_17 atlas.

The resulting 229 × 229 connectivity matrices were submitted to cov-STATIS, and the primary results regarding network connectivity and age effects on dedifferentiation and segregation scores are reported below. First, we examined the multivariate connectivity space of the nodes included in the Power atlas (Fig. 2). Similar to the original analyses, the multivariate space is characterized by differences between default vs. somato-motor/visual and dorsal attention/frontoparietal control vs. visual networks.

Next task differentiation scores were computed which describe the similarity of connectivity patterns for each Power network across cognitive domains. As in the original analysis, task differentiation for fronto-parietal control, ventral attention (which overlaps with Schaefer's temporoparietal network), salience and cingulo-opercular networks (which overlaps with Schaefer's salience/ventral attention A) decreased in older ages (Fig. 3). Although not significant, the negative effect of age on default differentiation (*r* = –.13, *p* = .132) and the non-linear age effect on the somato-motor network (r^2^ = .07*; p* = .115) trended in the same direction as the original findings. The Power somato-motor network is primarily motor and somato-motor cortex whereas the Schaefer somato-motor A and B networks include both motor/somato-motor and auditory cortices, which could explain the failure to replicate this specific finding.

Next, we examined network segregation for the Power atlas which described how much a network was connected to itself versus other networks. Age-related decreases in network segregation were replicated for fronto-parietal control, default mode, auditory (similar to Schaefer's somato-motor B subnetwork), cingulo-opercular (similar to Schaefer's salience/ventral attention A subnetwork), and dorsal attention networks (Fig. 4A). Age-related increases in network segregation were replicated for the somato-motor network (similar to Schaefer's somato-motor A subnetwork; Fig. 4B). We had also originally reported an age-related increase in Schaefer's salience/ventral attention B subnetwork, but this finding was not replicated in the most similar Power network (salience). However, the Power salience network includes nodes that overlap with other Schaefer networks (e.g., Default B, salience/ventral attention A; Table 2) that showed age-related increases which may account for this difference in findings between the two analyses.

#### 1.1.2 Schaefer 200 atlas analyses and results

In general, the Schaefer 200 parcel version of the 17-network atlas defines networks in a way similar to the 100 parcel atlas, but with smaller, more fine-grained parcels; however there are minor differences. For example, the 200 node 17 network atlas includes left and right inferior parietal in default A (yellow parcel) but the 100 node 17 network only includes right inferior parietal lobule (Fig. 5).

Using CONN, functional time courses for each task condition were extracted from the 200 parcels nodes that comprised 17 networks. The resulting 200 × 200 connectivity matrices were submitted to cov-STATIS, and the results regarding network connectivity and age effects on dedifferentiation and segregation scores are reported here. First, we examined the multivariate connectivity space (Fig. 6). Similar to the original analyses, the multivariate space is characterized by differences between limbic and default vs. somato-motor and dorsal attention/control vs. visual networks.

Next, task differentiation scores were computed which describe the similarity of connectivity patterns for each network. As in the original analyses, increased age was associated with decreased task differentiation for control, default, and salience/ventral attention (Fig. 7). The non-linear effect of age on somato-motor differentiation also replicated. However, the negative effect of age on the temporo-parietal network was not replicated (*r* = –.14, *p* = .105), although the finding was in the same direction. Compared to the 100 parcel version of this atlas, the 200 parcel atlas also includes regions of the temporo-parietal junction which may account for this difference in findings.

Next, we examined network segregation for the results using the Schaefer 200 atlas. As in the original analyses, older age was associated with decreased segregation for control, default, somato-motor B, and dorsal attention networks and increased segregation of somato-motor A network (Fig. 8A). The original finding of a negative effect of age on salience/ventral attention A (*r* = –.13, *p* = .127) and a positive effect of age on salience/ventral attention B (*r* = –.01, *p* = .869) segregation did not replicate with the more fine-grained 200 parcel atlas (although it trended in the same direction for the A subnetwork). The weakened network segregation findings for the salience/ventral attention sub-networks suggest that the age effects may not be homogenous across the individual regions of these networks.

## 2 Experimental Design, Materials and Methods

### 2.1 Experimental design

This dataset includes 144 participants, ages 20–86, recruited from the greater Toronto area for a study on functional activity during cognitive control (for more details on the larger study see [1,12]). Participants underwent *f*MRI while they completed three different tasks of cognitive control that were programed with E-prime 1.0 (scripts to replicate the experiment can be found here: https://osf.io/ceua6/). During scanning, participants lied supine on the scanner bed with a mirror in front of their face that reflected a computer screen that presented the experimental stimuli. Participants had earplugs in and headphones on to block out scanner noise, and a microphone allowed for communication with the experimenter between scans. Participants held a response box in their right hand and responded to stimuli on the screen with their index and middle fingers (specific finger responses were counterbalanced across participants). For all tasks, letters were presented in courier new font in the center of the computer screen on a dark gray background (see [12] Fig. 1 for a schematic of each task).

Inhibition and initiation were measured with a go/no-go paradigm in which participants were presented with a series of uppercase letters and told to respond (i.e., “go”) when they saw the letter “X” and not respond (i.e., “no-go”) for all other letters. No-go stimuli (i.e., non-Xs) were randomly drawn from a pool of 20 other letters: A, B, C, D, E, F, G, H, I, J, L, M, N, O, P, Q, R, S, T, and U. The task was separated into an “inhibition” block in which there were more go trials than no-go trials (120 go, 40 no-go) and a shorter “initiation” block in which there were more no-go trials than go trials (20 go, 60 no-go). The order of these two blocks was randomized across participants. Letter stimuli were presented for 400 ms with an average interstimulus interval (fixation cross) of 1200 ms that was randomly jittered between 900 and 1500 ms. The total time for the go/no-go task was 6 minutes 24 seconds.

Shifting was measured with a local-switching paradigm in which participants saw an upper- or lowercase vowel or consonant letter in the center of the screen and one of two cues above the letter to categorize the letter as either uppercase/lowercase or consonant/vowel. Letters were pseudo-randomly selected from the following set: A, a, E, e, I, i, U, u, F, f, M, m, R, r, T, and t. The cues and letters were presented in two different colors (blue and green) based on the judgement type. The cues were positioned above the central letter to the right and left, and the position of the cues and corresponding color were counterbalanced across judgment types. There was a total of 60 trials of which 30 were uppercase/lowercase judgments and 30 were consonant/vowel judgments. Trials were also ordered in such a way that half of the trials repeated the same judgment (e.g., vowel/consonant followed by vowel/consonant) and half of the trials switched judgment type (i.e., vowel/consonant followed by uppercase/lowercase). Letters were presented for 2000 ms with a mean interstimulus interval (fixation cross) of 4500 ms that was randomly jittered between 1500 and 7400 ms. The total time for the switching paradigm was 7 minutes 26 seconds.

Working memory was measured using a standard n-back paradigm with 0-, 1-, and 2-back loads in which participants saw a series of uppercase letters and had to respond if the letter was a “target” or “non-target”. For 0-back, the targets were “X” and all other letters were non-targets (pseudo-randomly selected from all other non-X letters). For 1-back, targets were letters that matched the previously presented letter within the sequence (pseudo-randomly selected from all non-X letters). For 2-back, targets were letters that matched the letter presented two positions back in the sequence (pseudo-randomly selected from all non-X letters). The task was organized into three blocks (one for each condition), and the order of the blocks was randomized. Within each block there were 30 target and 60 non-target trials. Letters were presented for 500 ms with an average interstimulus interval (fixation cross) of 1200 ms that was randomly jittered between 900 and 1500 ms. The total time for the working memory paradigm was 8 minutes 52 seconds.

All participants were scanned on the same Siemens Trio 3T magnet at Baycrest Health Sciences while they completed the three *f*MRI tasks of cognitive control. Blood-oxygen-level-dependent (BOLD) *f*MRI data were collected with a 12-channel head coil using an echo-planar imaging sequence with 40 axial slices acquired parallel to the anterior-posterior commissure with the following parameters: TR = 2,000 ms, TE = 27 ms, Flip Angle = 70°; FOV = 192 mm, 64 × 64 × 40 acquisition matrix; 3 mm^3^ isotropic voxels (with .5 mm gap). A total of 216 volumes were collected for the go/no-go task; 223 volumes collected for task switching; and 266 volumes collected for the n-back task. High resolution anatomical scans used for warping the BOLD images to MNI space were acquired with a T1-weighted MP-RAGE sequence in which 160 axial slices were collected with the following parameters: TR = 2000ms, TE = 2.63 ms, FOV = 256 mm; 192 × 256 × 160 acquisition matrix; 1 mm^3^ isotropic voxel.

### 2.2 fMRI preprocessing

Functional data for each task were preprocessed with a mix of AFNI functions as well as Octave and MATLAB scripts using the Optimizing of Preprocessing Pipelines for NeuroImaging software package (OPPNI; an overview and more details of the preprocessing pipeline can be found in [13]). The latest version of OPPNI software is available upon request (https://github.com/strotherlab/oppni). For the current dataset, a fixed pipeline for all participants was conducted with the following steps: (1) *3dvolreg* in AFNI for rigid-body alignment of the timeseries to correct for movement; (2) removal and interpolation of outlier volumes using Octave scripts; (3) *3dretroicor* in AFNI for correction for physiological (i.e., cardiac and respiratory) noise; (4) *3dTshift* in AFNI for slice timing correction; (5) *3dmerge* in AFNI spatial smoothing with a 6 mm smoothing kernel; (6) temporal detrending with in-house MatLab scripts; (7) regression of six motion parameter estimates (X, Y, and Z translation and rotation) on the timeseries with in-house MATLAB scripts; (8) regression of signal in tissue of no interest (white matter, vessels and cerebrospinal fluid) on the timeseries with in-house MATLAB scripts; and finally (9) warping to MNI space and resampling to 4 mm^3^ isotropic voxel.

### 2.3 Functional connectivity methods

Functional connectivity was computed for each of the three *f*MRI tasks using the CONN toolbox [14] in MATLAB. The primary results from the Schaefer100_17 atlas parcellation can be found in the related manuscript [1] and are also summarized here (Table 1). Two additional atlases were also used to replicate the primary findings: (1) an iteration of the network coordinates provided by Power and colleagues [4] that included 229 5 mm spherical nodes associated with 10 functional networks (Power229_10) and (2) the Schaefer [2] 200 parcel, 17 network atlas (Schaefer200_17) which is roughly equivalent to the 100 parcel atlas, but with smaller and more fine-grained parcels. See Figs. 1 and 5 for side-by-side comparisons of the different atlases.

Using CONN, task design (i.e., trial onsets) was first regressed out of the timeseries. Next, the BOLD signal in each voxel was converted to a percent signal change value by scaling the whole timeseries to the average timeseries value of that voxel. Percent signal change values were averaged across voxels within each of the nodes or parcels for the corresponding atlas. Finally, the timeseries for each node or parcel were correlated (using Pearson's correlation) to create a correlation matrix associated with each task condition. For the go/no-go task, timeseries correlations were computed separately for the “inhibition” (i.e., more “go”) and “initiation” (i.e., more “no-go”) blocks. For task switch, the entire timeseries was used to compute the correlation. For n-back, the timeseries correlations were computed separately for each working memory load block (0-, 1-, and 2-back). This resulted in the current dataset which includes six correlation matrices per participant representing the functional connectivity during different conditions of cognitive control (inhibition, initiation, switching, 0-, 1-, and 2-back working memory) for each of the three brain atlases. All functional connectivity matrices are square, symmetric, positive semidefinite matrices (of size 100 × 100, 229 × 229, or 200 × 200 depending on the atlas used) with a diagonal of 1 and values ranging between -1 and 1.

## Ethics Statement

Participants’ informed consent was obtained in accordance with protocol approved by the Research Ethics Board at Baycrest Health Sciences Center.

## CRediT Author Statement

**Jenny R. Rieck:** Conceptualization, Methodology, Formal analysis, Investigation, Data curation, Writing – original draft, Visualization, Project administration; **Giulia Baracchini:** Investigation, Data curation, Writing – review & editing, Project administration; **Daniel Nichol:** Data curation, Software; **Hervé Abdi:** Conceptualization, Methodology, Software, Writing – review & editing, Supervision; **Cheryl L. Grady:** Conceptualization, Investigation, Resources, Writing – review & editing, Supervision, Project administration, Funding acquisition.

## Declaration of Competing Interest

The authors declare that they have no known competing financial interests or personal relationships which have or could be perceived to have influenced the work reported in this article.
